# Transcriptional and Functional Analysis of Bifidobacterium animalis subsp. lactis Exposure to Tetracycline

**DOI:** 10.1128/AEM.01999-18

**Published:** 2018-11-15

**Authors:** Wesley Morovic, Paige Roos, Bryan Zabel, Claudio Hidalgo-Cantabrana, Anthony Kiefer, Rodolphe Barrangou

**Affiliations:** a Genomics & Microbiome Science, DuPont Nutrition & Health, Madison, Wisconsin, USA; b Genomics Laboratory, DuPont Pioneer, Johnston, Iowa, USA; c Department of Food, Processing and Nutrition Sciences, North Carolina State University, Raleigh, North Carolina, USA; d Probiotic Development, DuPont Nutrition & Health, Madison, Wisconsin, USA; Goethe University Frankfurt am Main

**Keywords:** Bifidobacterium animalis subsp. *lactis*, antibiotic resistance, tetracycline, RNA sequencing, genomics, droplet digital PCR, ATP luminescence

## Abstract

Commercial probiotic bacteria must be tested for acquired antibiotic resistance elements to avoid potential transfer to pathogens. The European Food Safety Authority recommends testing resistance using microdilution culture techniques previously used to establish inhibitory thresholds for the Bifidobacterium genus. Many Bifidobacterium animalis subsp. lactis strains exhibit increased resistance to tetracycline, historically attributed to the ribosomal protection gene *tet*(W). However, some strains that harbor genetically identical *tet*(W) genes show various inhibition levels, suggesting that other genetic elements also contribute to observed differences. Here, we adapted several molecular assays to confirm the inhibition of B. animalis subsp. *lactis* strains Bl-04 and HN019 and employed RNA sequencing to assess the transcriptional differences related to genomic polymorphisms. We detected specific stress responses to the antibiotic by correlating ATP concentration to number of viable genome copies from droplet digital PCR and found that the bacteria were still metabolically active in high drug concentrations. Transcriptional analyses revealed that several polymorphic regions, particularly a novel multidrug efflux transporter, were differentially expressed between the strains in each experimental condition, likely having phenotypic effects. We also found that the *tet*(W) gene was upregulated only during subinhibitory tetracycline concentrations, while two novel tetracycline resistance genes were upregulated at high concentrations. Furthermore, many genes involved in amino acid metabolism and transporter function were upregulated, while genes for complex carbohydrate utilization, protein metabolism, and clustered regularly interspaced short palindromic repeat(s) (CRISPR)-Cas systems were downregulated. These results provide high-throughput means for assessing antibiotic resistances of two highly related probiotic strains and determine the genetic network that contributes to the global tetracycline response.

**IMPORTANCE** Bifidobacterium animalis subsp. lactis is widely used in human food and dietary supplements. Although well documented to be safe, B. animalis subsp. *lactis* strains must not contain transferable antibiotic resistance elements. Many B. animalis subsp. *lactis* strains have different resistance measurements despite being genetically similar, and the reasons for this are not well understood. In the current study, we sought to examine how genomic differences between two closely related industrial B. animalis subsp. *lactis* strains contribute to different resistance levels. This will lead to a better understanding of resistance, identify future targets for analysis of transferability, and expand our understanding of tetracycline resistance in bacteria.

## Abstract (precis)

Bifidobacterium animalis subsp. lactis is widely used in human food and dietary supplements. Although well documented to be safe, B. animalis subsp. *lactis* strains must not contain transferable antibiotic resistance elements. Many B. animalis subsp. *lactis* strains have different resistance measurements despite being genetically similar, and the reasons for this are not well understood. In the current study, we sought to examine how genomic differences between two closely related industrial B. animalis subsp. *lactis* strains contribute to different resistance levels. This will lead to a better understanding of resistance, identify future targets for analysis of transferability, and expand our understanding of tetracycline resistance in bacteria.

## INTRODUCTION

Bifidobacterium animalis subsp. lactis is a Gram-positive commensal species with a long history of safe use in food (1). B. animalis subsp. *lactis* is often used as an ingredient in yogurt and as a main component of probiotic dietary supplements (2, 3). Probiotics are defined as live microorganisms that, when administered in adequate amounts, confer a health benefit on the host (4, 5). Indeed, clinical studies assessing supplementation with B. animalis subsp. *lactis* have revealed probiotic effects, such as improved gastrointestinal transit and comfort (6–8), immune modulation (9, 10), and control of abdominal fat mass (11). More recently, microbiome analysis by DNA sequencing has demonstrated that B. animalis subsp. *lactis* also modulates gut and colorectal cancer-associated microbiota (12, 13). Genome sequencing of B. animalis subsp. *lactis* strains has determined genetic modes of action for various cellular functions and showed this subspecies to be somewhat monomorphic (14–17). Although all B. animalis subsp. *lactis* strains characterized so far are generally considered safe for consumption and show no toxic effects during *in vivo* animal studies (1), many exhibit tetracycline resistance, which is typically attributed to a *tet*(W) gene (18, 19). Furthermore, many strains have mobile genetic elements adjacent to the *tet*(W) gene, which may contribute to horizontal gene transfer, as demonstrated previously (20). Attempts to force translocation of *tet*(W) by conjugation have failed, which suggests that that this genetic combination does not pose a major transfer threat and may therefore be safe for consumption (18, 21). Interestingly, strains of B. animalis subsp. *lactis* with genetically identical *tet*(W) and transposase genes have different inhibition levels (18, 22, 23).

Tetracycline antibiotics were first developed in the 1940s and are used for many human ailments, such as pneumonia, cholera, and malaria (24), as well as in agriculture for growth promotion (25). Tetracyclines are a family of broad-spectrum, bacteriostatic drugs that prevent the aminoacyl-tRNA from binding to the A site on the 30S ribosome, thus inhibiting the elongation step in ribosomal protein synthesis (24, 26). Expanded- and broad-spectrum tetracycline families were developed to broaden the range of targets and circumvent resistance (27). There are four known tetracycline resistance mechanisms, as follows: factor-assisted ribosomal protection, point mutation of the ribosome, removal of the drug by efflux, and enzymatic inactivation of the drug (26–28). The *tet*(W) gene in B. animalis subsp. *lactis* is a GTPase that protects the ribosome by modifying the tetracycline target binding site (29). To test inhibition levels, the European Food Safety Authority (EFSA) recommends growing an individual strain in a concentration gradient of antibiotic until visible growth is inhibited (30). Here, we use microbiological and molecular methods to examine strain-specific responses to tetracycline in B. animalis subsp. *lactis* Bl-04 and HN019, commercial probiotic strains that have been confirmed to have few genomic differences, while likely having phenotypic differences (15).

## RESULTS

### *In vitro* antibiotic testing.

**FIG 1:**
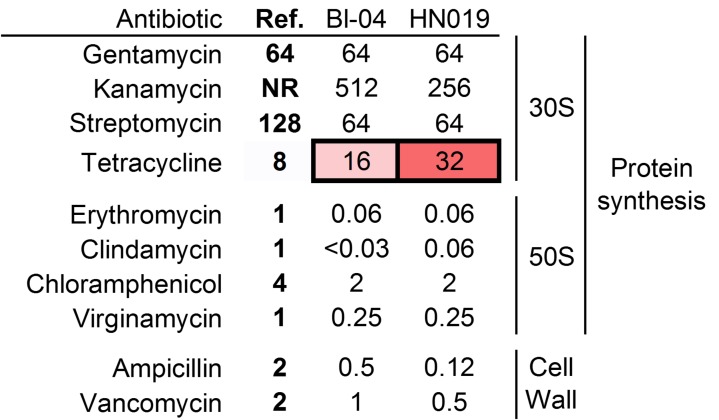
MIC profiles compared to the breakpoints for various antibiotics. Concentrations are in μg/ml. Boldface values are the thresholds for each antibiotic. Values in red are above the thresholds for bifidobacteria. Ref., threshold level for antibiotic. NR, testing was not required.

B. animalis subsp. *lactis* Bl-04 and HN019 were tested against the full complement of antibiotics outlined by the EFSA (Fig. 1). Interestingly, both strains have similar MIC levels for gentamicin and streptomycin, which target the 30S ribosome, at or below the recommended threshold. However, the MICs of the strains were above the recommended tetracycline threshold set for bifidobacteria (8 μg/ml), being 16 μg/ml and 32 μg/ml for Bl-04 and HN019, respectively. Furthermore, resistance to kanamycin was higher in Bl-04, although there is no recommended threshold. The strains were not above the threshold for any of the other antibiotics tested. These results demonstrate the specificity of resistance to individual drug molecules within strains.

**FIG 2:**
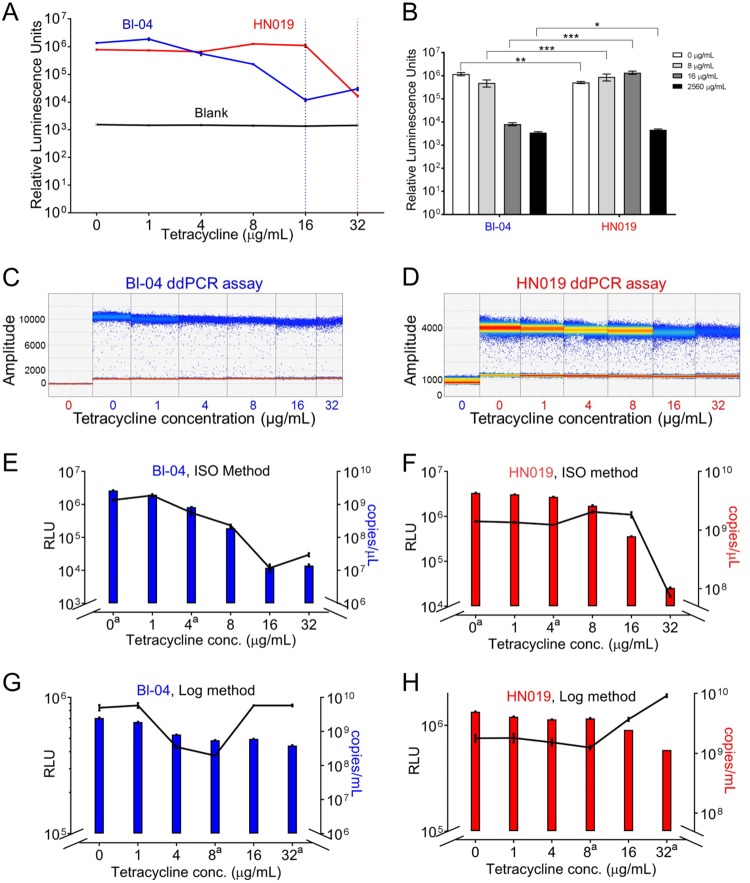
Assay of tetracycline response using ATP luminescence and droplet digital PCR. (A) ATP quantification using the microdilution procedure, with the dotted vertical lines showing the MIC levels for both strains. (B) The difference in ATP response between the two strains at the MIC thresholds (*, *P* < 0.01; **, *P* < 0.001; ***, *P* < 0.0001; paired *t* test). Droplet digital PCR results for the (C) Bl-04 and (D) HN019 assays show number of droplets per amplitude with a quantitative heat map applied. Sample colors in the *x* axes denote Bl-04 (blue) and HN019 (red) by tetracycline concentration. Droplet digital PCR assay results are shown as colored bars for (E) Bl-04 and (F) HN019 compared to ATP concentration (black lines). The experiment was repeated using acute exposure to tetracycline for (G) Bl-04 and (H) HN019. The *y* axes have been adjusted to better show overall correlation. ^a^Conditions used for the RNA-seq experiments.

ATP concentrations were assayed to quantify any potential nonvisible growth over a range of tetracycline concentrations (Fig. 2A). ATP concentration was measured, as it is indicative of cellular metabolism and enzymatic processes (31). The tetracycline dilution series was aligned with the Bioo tetracycline detection assay standards to ensure correct amounts (Fig. S1). Both strains showed overall decreases in ATP levels at the respective MICs; however, the ATP levels did not reach the levels of the negative control. The strains were further tested at 8 and 16 μg/ml tetracycline and were significantly different at each concentration (2-sample *t* test; *P* < 0.001) (Fig. 2B). Surprisingly, the ATP concentration in HN019 increased with added tetracycline. Because ATP-activated efflux is a key resistance factor, we hypothesized that the increase in HN019 represents a stress response rather than growth. Digital PCR has recently been demonstrated to rapidly and accurately quantify probiotic cells (32), and it was used to quantify each strain separately (Fig. 2C and D). Comparing droplet digital PCR (ddPCR) to ATP concentrations (Fig. 2E and F) showed the concentration of both strains declining with increased tetracycline, which correlated with the ATP concentration for Bl-04 (*P* = 0.01) but not with that for HN019 (*P* = 0.569). We further hypothesized that measuring an acute exposure of the strains to tetracycline would demonstrate resistance more relevant for *in vivo* conditions, as the ISO method has a very small starting concentration of cells. The alternative method to the ISO procedure (denoted as LOG experiments) showed that ATP concentrations decreased in both strains until 8 μg/ml, followed by drastic increases in ATP compared to the slope of the ddPCR (Fig. 2G and H). Interestingly, after the initial decrease, the ATP concentrations peaked at 16 μg/ml for Bl-04 and 32 μg/ml for HN019, which are their respective MICs using the ISO method. This suggested that there are different cellular responses to tetracycline, and it might reflect normal growth behavior rather than persistence with a stress response at higher antibiotic concentrations.

### Genome comparison.

**FIG 3:**
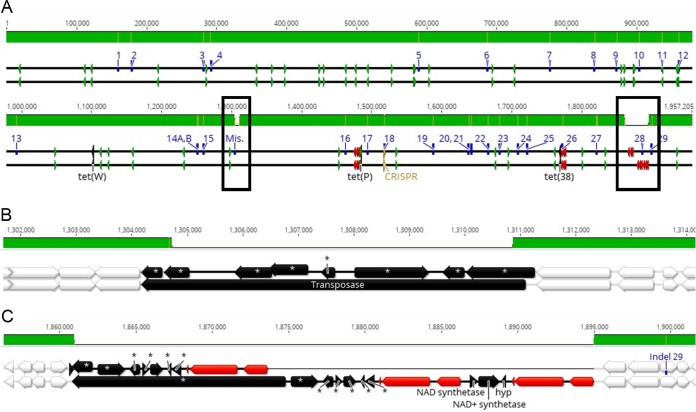
Comparative genomics of B. animalis subsp. *lactis* Bl-04 and HN019. The HN019 genome (top black line) is aligned to Bl-04 (bottom black line) using progressiveMauve. The top green bar shows identity, with yellow and white areas showing polymorphisms and deletion events, respectively. The sequence lines show various annotations, as follows: blue, indels from Fig. 6; green, tRNAs; red, rRNAs; gold, CRISPR-Cas. The whole-genome alignment is divided into two continuous indel sequence lines (A) and zoomed in to the (B) possibly misassembled and (C) indel 28 regions. Annotations with asterisks are present elsewhere in the opposite genome. White annotations are identical between the strains. Unique gene names are annotated.

A hybrid sequencing approach for B. animalis subsp. *lactis* HN019 yielded a complete genome of 1,935,423 bp with an overall G+C content of 60.5%, and rapid annotation using subsystem technology (RAST) annotation predicted 1,620 coding sequences and 61 RNAs. While previous studies have compared the genomes of various B. animalis subsp. *lactis* strains, HN019 has been assessed either by targeted sequencing of specific genomic regions, or using a draft sequence (14, 15). Our genome sequence of HN019 showed 97.9% nucleotide pairwise identity to that of Bl-04 and was highly similar in structure and content (Fig. 3A). There were, however, genetic differences between the strains at 29 distinct genomic locations (Table S1), which were assessed using targeted PCR (Table S1). Previously reported deletions in a long-chain-fatty-acid–coenzyme A (CoA) ligase gene and clustered regularly interspaced short palindromic repeat (CRISPR) spacer region were confirmed (15). Insertion/deletion (indel) locations 22 and 23 were confirmed to be errors in the Bl-04 genome, and indels 26 and 28 could not be confirmed by PCR due to repeated nucleotide regions. There was a large difference at Bl-04 position 1304794, annotated as a transposase (BALAC_RS05595) (Fig. 3B). The HN019 genome instead encoded an ABC transporter and alpha-glucosidase that are located near indel 28 in the Bl-04 genome. Furthermore, the Bl-04 genome showed an additional rRNA operon near the same indel. Because repeated elements like transposons and rRNAs are notoriously difficult to assemble, especially without long-read sequencing technology (33), we assumed the Bl-04 genome had an assembly error and did not include genes from the misassemblies in the downstream indel analysis. Interestingly, the Bl-04 genome encoded an NAD synthetase, an NAD^+^ synthetase, and a hypothetical protein (predicted COG3077 DNA damage-inducible protein J) starting at position 1868346 in the Bl-04 genome that are not in the HN019 genome, although there was a different NAD synthetase in the HN019 genome at position 1244980 (Fig. 3C). The new HN019 assembly also showed two small intergenic insertions at positions 1721502 and 1820496 in the HN019 genome. Indels 14A and 14B occur in the same gene, and 14B alters a predicted restriction site found in B. animalis subsp. *lactis* (34). Comparing functional scenarios using RAST showed the same results in most categories, and all differences were due to the above polymorphisms. The additional stress response element was an error in the RAST prediction.

Reannotating both strains with RAST revealed seven genes related to antibiotic resistance, as follows: three for tetracycline, including *tet*(W), and four for fluoroquinolones. The genomes were compared to the Antibiotic Resistance Genes Database (ARDB), Comprehensive Antibiotic Resistance Database (CARD), and Antibiotic Resistance Gene-ANNOTation (Arg-Annot) using a blastx search, but no additional resistance genes met the 80% nucleotide identity threshold. The *tet*(W) gene and two putative tetracycline resistance genes had 100% DNA sequence identity between the two strains, and neither putative gene was in proximity (within 5 kb) to known mobile genetic elements. Both tetracycline resistance genes were further analyzed for protein function relative to *tet*(W), as antibiotic resistance genes share similar functionality with nonresistance genes that may be difficult to distinguish (35). The gene at Bl-04 position 1479751 was originally annotated as a translation elongation factor for GTPases (EF-G; locus tag BALAC_RS06365), and had a Tet(M) domain similar to that of the *tet*(W) gene. This is likely due to elongation factors binding to the same region of the ribosome as resistance proteins. The closest match to this gene in the ARDB was *tet*(P) from Clostridium perfringens, with a 30.1% amino acid match, which also has a 37.8% identity to the *tet*(W) amino acid sequence. The gene with locus tag BALAC_RS07425 at Bl-04 position 1763551 is a transport permease with amino acid regions that match antibiotic resistance transporters. The amino acid sequence is 99.8% identical to that of *tet*(36) in B. animalis subsp. *lactis* AD011. A *tetR* tetracycline repressor gene (BALAC_RS07655) was also homologous between the two strains. Other nonantibiotic resistance permeases are present in both strains, and efflux transport is a widespread feature, as there are 31 other membrane transport genes encoded in that genome (Table 1).

**TABLE 1:** Metabolic responses to the tetracycline experiments

RAST category	B. lactis strain^*a*^:													
	Bl-04							HN019						
	Annotated genes^*b*^	ΔISO_4		ΔLOG_8		ΔLOG_32		Annotated genes^*c*^	ΔISO_4		ΔLOG_8		ΔLOG_32	
		↓	↑	↓	↑	↓	↑		↓	↑	↓	↑	↓	↑
Amino acids and derivatives	200			1	17	13	24	200		1	1	17	11	18
Carbohydrates	158			15	12	41	23	163			20	12	59	9
Cell division and cell cycle	24			1		2		24					1	
Cell wall and capsule	56			4		16	2	56			2		17	2
Cofactors, etc.	80				11	2	18	76				11	2	13
DNA metabolism	56			2		7	4	55			3		7	3
Dormancy and sporulation	1							1						1
Fatty acids, lipids, and isoprenoids	34				6	1	6	34			5	6	1	6
Iron acquisition and metabolism	0							0						
Membrane transport	31				10	4	16	31				10	4	14
Metabolism of aromatic compounds	3							3						
Miscellaneous	13				1	1	1	13				1	7	1
Motility and chemotaxis	5			1	1	2	1	5			1	1	2	
Nitrogen metabolism	8							8						3
Nucleosides and nucleotides	67			5		6		67			2		8	1
Transposable elements, etc.	0							0						
Phosphorus metabolism	24					7		24			1		6	
Photosynthesis	0							0						
Potassium metabolism	11			1		2	6	11			2		3	
Protein metabolism	176			1		30	4	176			4		12	6
Regulation and cell signaling	12			1	1	6	1	11			1	1	3	
Respiration	11					7		11					7	1
RNA metabolism	63				1	1	6	63				1	1	4
Secondary metabolism	2					2		1					2	
Stress response	30		1	3	1	5	4	31			1	1	5	6
Sulfur metabolism	18			2	1	2	7	18			3	1	2	8
Virulence, disease, and defense	28			1	3	3	4	28			2	3	4	2
Not in subsystem	867	31	27	103	122	219	286	872	12	1	134	108	234	289

a The numbers of genes in each metabolic category that are significantly upregulated (↑) or downregulated (↓) are shown by strain and by results of each experiment compared to those of the control experiment (ISO_0).

b Total number of genes annotated by RAST in the B. lactis Bl-04 genome.

c Total number of genes annotated by RAST in the B. lactis HN019 genome.

### Transcriptional response to tetracycline.

**FIG 4:**
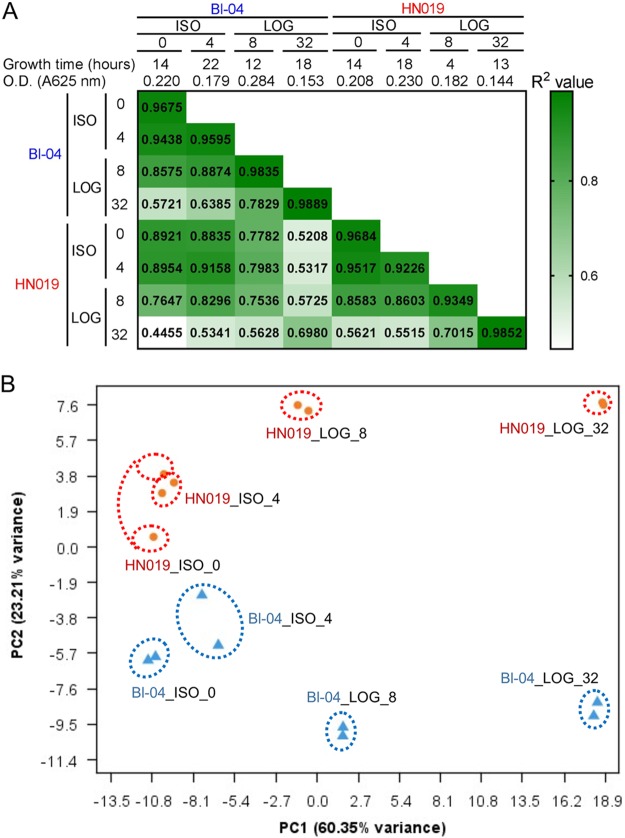
Overview of the RNA sequencing results. (A) The test conditions and *R*^2^ correlations between different sample sets are shown with the heat map. Replicates are shown in the diagonal cells that match experiment names. (B) Principal-coordinate analysis shows the variation between strains determined using the DESeq2 method for Bl-04 (blue triangles) and HN019 (orange circles).

**FIG 5:**
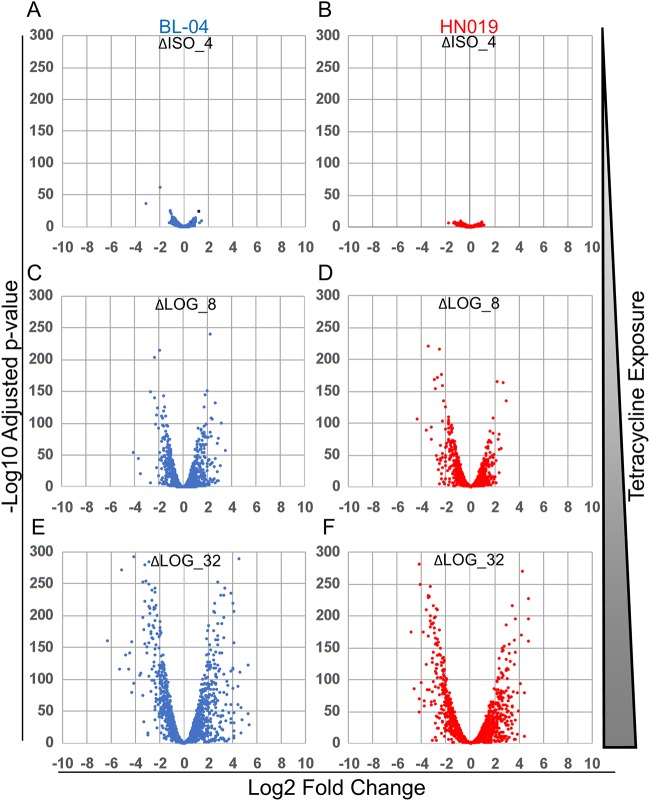
Volcano plots of expression differences between Bl-04 and HN019 during different tetracycline exposures. Experimental names compared to the control ISO_0 experiment with no tetracycline are shown at the top of each plot and are as follows: (A) Bl-04 ΔISO_4, (B) HN019 ΔISO_4, (C) Bl-04 ΔLOG_8, (D) HN019 ΔLOG_8, (E) Bl-04 ΔLOG_32, and (F) HN019 ΔLOG_32. Negative log change represents higher expression in the control (ISO_0), and positive log change indicates higher expression in the respective treatments. Results are shown for Bl-04 (blue) and HN019 (red).

RNA transcript sequencing yielded an average of 6,594,563 paired-end reads per experiment. Although single reads were generated for each replicate, only paired-end reads were included in subsequent analysis. All read sets from both strains were mapped to the B. animalis subsp. *lactis* Bl-04 genome to directly analyze differences between the strains, with the Bl-04_ISO_0 experiment as the overall control. The read assemblies confirmed the PCR test findings, with indels 22, 23, and 27 incorrect in Bl-04. Reads could not be distinguished between the different RNA gene operons, so indel 26 could not be analyzed. None of the HN019 experiments resulted in reads for the NAD synthetase operon (indel 28), confirming the absence of the associated genes. The average correlation of the biological replicates was *R*^2^ = 0.9638, with the lowest correlation being *R*^2^ = 0.9226 for HN019_ISO_4 and the highest being *R*^2^ = 0.9889 for Bl-04_LOG_32 (Fig. 4A). Analysis of all replicates by principal-coordinate analysis (PCA) (Fig. 4B) showed replicates clustering better with the higher concentrations of tetracycline. Importantly, the experiments separated by concentration along the *x* axis and by strain along the *y* axis, indicating that antibiotic effect accounts for much of the observed differences, followed by strain. The overall gene expression differed from that of the control with increased tetracycline exposure (Fig. 5).

**FIG 6:**
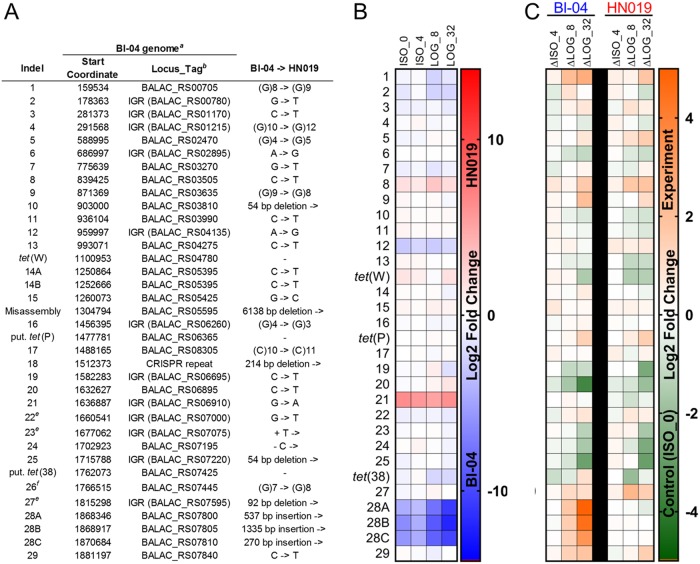
Expression differences of the different polymorphic regions. (A) Overview of polymorphic regions. ^a^Indel references are made to the Bl-04 genome. ^b^Locus tags in parenthesis are genes associated with intergenic indels that were assessed in the expression data. ^e^Indels are errors in the Bl-04 genome. ^f^Indel could not be confirmed by PCR or transcript read analysis. (B) Expression differences at each locus between the strains showing upregulation in Bl-04 (blue) or HN019 (red). (C) Expression differences at each locus within the strains show upregulation in the control ISO_0 (green) or treatments (orange).

The assembly of reads to a single genome allowed for direct comparison of expression levels between experiments for each gene (Fig. 6). We first assessed the polymorphic differences between the two strains grown in ISO media without tetracycline (labeled ISO_0) to establish expression changes not due to tetracycline exposure. Genetic differences in intergenic regions were analyzed for potential promoters, and then the flanking genes were analyzed for transcription differences. Indels 2, 12, and 28 were significantly upregulated in Bl-04 cultures without tetracycline compared to in HN019 cultures. Indel 2 is a single-nucleotide transversion upstream of *yihS*, which isomerizes a range of sugars. Indel 12 is in the intergenic region upstream of a hypothetical gene related to a PAC2 family proteasome assembly chaperone and downstream of a putative promoter.

**FIG 7:**
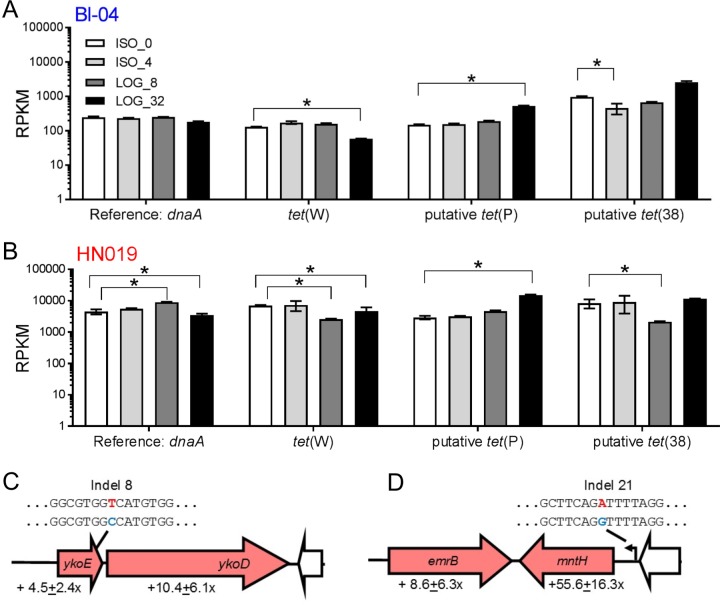
Differential expression of the three key genetic regions. Comparison of the raw reads per kilobase million (RPKM) for the three tetracycline resistance genes in (A) Bl-04 and (B) HN019 (*, adjusted *P* < 0.05 and fold change of >2×). Genomic representations of the key polymorphic regions (C) indel 8 and (D) indel 21, shared between the two strains, are represented by block arrows. Arrows are colored as follows: red, upregulated in HN019; white, identical genes; and black, putative promoter. Nucleotide identities for each strain are denoted by color, with Bl-04 and HN019 being blue and red, respectively.

Indels 8 and 21 and *tet*(W) were significantly upregulated in HN019 compared to those in Bl-04 in experiment ISO_0 (Fig. 7). Indel 8 is a single-nucleotide polymorphism (SNP) that causes an alanine to valine mutation in *ykoE*, which transports hydroxymethylpyrimidine (HMP) and thiamine (vitamin B_1_). The adjacent ABC-binding protein is also upregulated in HN019. Indel 21 is in a putative promoter that may upregulate the upstream transport gene *mntH* (manganese, iron, and other metal transport), as well as an *emrB* multidrug transport gene previously annotated as a hypothetical gene. Interestingly, the *mntH* and *emrB* genes are oriented in opposite directions, yet similarly upregulated. Without the addition of tetracycline, *tet*(W) expression is higher in HN019, although there are no proximate genetic differences. This shows that *tet*(W) is constitutively expressed and may have further roles in general cell function. Furthermore, HN019 cultures generally reached the target optical density quicker than did Bl-04 cultures during each experiment (Fig. 4A), which indicates that these genetic polymorphisms provide phenotypic benefits.

The ISO_0 and ISO_4 experiments for both Bl-04 and HN019 were correlated within the range of the biological replicates (Bl-04 *R*^2^ = 0.9675; HN019 *R*^2^ = 0.9517). The expression profiles between the two strains correlated more during ISO_4 (*R*^2^ = 0.9158) than under normal growth conditions in ISO_0 (*R*^2^ = 0.9675). Twenty-seven genes were significantly upregulated in Bl-04 during exposure to ISO_4, seven of which are involved with transmembrane transport. Only one gene was assigned to a RAST category, the cold shock gene *cspA*, which was upregulated in the LOG experiments as well (Table 1).

Other genes, such as two *cas* genes in Bl-04, were significantly downregulated when there were subinhibitory levels of tetracycline, and all of the *cas* genes in the system were downregulated throughout the LOG tetracycline exposures (Fig. S4). The *tet*(W) gene expression only increased by 0.64-fold (adjusted *P* = 0.023). The putative *tet*(P) expression did not change and the putative *tet*(36) expression significantly decreased. Only two genes were significantly upregulated in HN019 during ISO_4, an isomerase involved in leucine biosynthesis and a transport component gene. Although *tet*(W) expression in HN019 was significantly higher in the ISO_0 experiment than Bl-04, it was not increased during the ISO_4 experiment. Indels 8 and 21 were also upregulated in HN019, which suggests contribution to tetracycline resistance as well as typical growth.

The *in vitro* testing showed Bl-04 and HN019 to have different tetracycline response thresholds, so results of the LOG_8 trial in HN019 were expected to be more related to those of the ISO trials, signifying resistance, while results of the LOG_8 trial in Bl-04 was expected to be more like those of LOG_32, since it had hit the threshold for the stress response. The PCA plot showed evidence of this, but the LOG_8 trials for the strains did not vary drastically (Fig. 4B). Nonetheless, the expression profiles for the higher concentrations were drastically different than those for the lower concentrations (Fig. 5).

The *tet*(W) expression decreased, while *tet*(P) and *tet*(36) expression increased with LOG exposure (Fig. 7A and B), which indicates that the latter two genes may provide better protection from the antibiotic to preserve cells in extreme exposure. Between the two strains, indels 19 and 20 showed expression differences only in the LOG experiments, which indicates tetracycline-dependent expression changes. Indel 19 is an intergenic SNP downstream of a galactokinase *galK* (Kyoto Encyclopedia of Genes and Genomes enzyme entry EC 2.7.1.6) that is upregulated in HN019 during LOG_8. Indel 20 is a SNP in the galactosyl transferase *cpsD* (EC 2.7.8.6) that was expressed higher in HN019 in LOG_8 and LOG_32. Indels 8 and 21 are again upregulated in HN019 for the LOG experiments, showing that both polymorphisms affect static expression of the associated genes (Fig. 7C and D).

Genes from many different metabolic categories were significantly downregulated and upregulated in both strains during the LOG experiments (Table 1); however, most differences were seen in amino acid and carbohydrate transport and metabolism. Bl-04 and HN019 had upregulated genes for methionine biosynthesis. Alternatively, genes involved in glutamine, glutamate, aspartate, asparagine, threonine, homoserine, glycine, and alanine biosynthesis were downregulated in the LOG experiments for the two strains.

Carbohydrate utilization was also affected in the LOG experiments. Genes involved with lactose and galactose uptake and utilization were downregulated in LOG_8 and LOG_32, which is interesting, considering indels 19 and 20. Genes for fructoooligosaccharides, raffinose, maltose, and maltodextrin uptake were also downregulated. Interestingly, seven genes involved in the pentose phosphate pathway are downregulated in the LOG_32 experiments of both strains. A key indicator of metabolic status is the fructose-6-phosphate phosphoketolase gene *xfp* (EC 4.1.2.22), which is essential for saccharolytic fermentation as part of the “bifidus shunt” (37). The *xfp* gene is significantly downregulated in the two strains in the LOG_32 experiment. Conversely, genes involved with xylose utilization were upregulated in both strains across the LOG experiments, although xylose is not in either the MRS or ISO-Sensitest media. The cell wall and capsule metabolic category included genes downregulated in both strains, specifically in dTDP-rhamnose synthesis and rhamnose-containing glycans.

**FIG 8:**
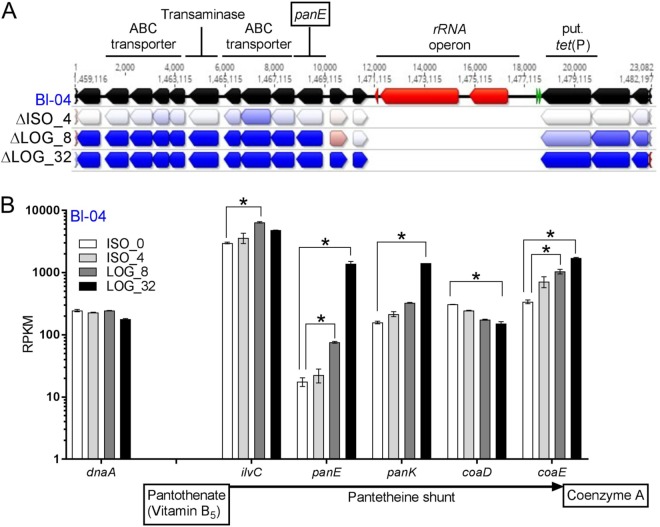
Overview of upregulated gene cluster associated with coenzyme A biosynthesis in B. animalis subsp. *lactis* Bl-04. (A) The genetic region was annotated in the Bl-04 genome and colored as follows: black, coding sequences; red, rRNAs; and green, tRNAs. The arrows below each gene represent a log_2_ ratio fold change between ISO_4, LOG_8, and LOG_32 compared to ISO_0 in descending order, with heat coloring showing downregulation (red) and upregulation (blue) with tetracycline. (B) The raw RPKM expression in Bl-04 for various genes involved in coenzyme A biosynthesis from pantothenate. *, adjusted *P* < 0.05 and fold change of >2×. Expression of *dnaA* is shown for reference.

Of the cofactors, genes involved in riboflavin (vitamin B_2_), NAD, and coenzyme A (CoA) production were all upregulated in the LOG experiments in both strains. Interestingly, there is a cluster of 12 genes that are significantly upregulated in the LOG experiments upstream of an rRNA operon, two tRNAs (valine and glycine), and the putative *tet*(P) gene (Fig. 8A). One of the genes in the cluster is 2-dehydropantoate 2-reductase *panE* (EC 1.1.1.169), which is key in the biosynthesis of coenzyme A. Other genes that make up the pantetheine shunt are present in both strains, and most are similarly upregulated during the LOG experiments (Fig. 8B). Of the 12 genes clustered together, at least seven are involved with transport, one set for glutamate/aspartate and one for various metal ions. BLAST analysis failed to find the pantothenate transport genes *panT* and *panF* in the two strains, and neither have been reported in bifidobacteria, although there were transporter genes adjacent to the *ilvC*, *panE*, and *panK* genes.

## DISCUSSION

Bacteria rapidly evolve countermeasures to resist increasingly overused and misused antibiotics, creating widespread resistance years after initial drug introduction (36, 38). The molecular benefits of developing resistance mechanisms far outweigh the costs (39, 40), so bacteria are constantly under pressure to acquire resistance elements (41). Monitoring antibiotic resistance and transferability is a concern in the food and dietary supplement industries to prevent the risk of industrial bacteria becoming reservoirs of resistance genes that can be transferred to pathogens (30, 42). Such products are most often tested using the ISO method for dairy products, which requires MIC measurements be compared to those for a representative species or an overall genus, as is the case of Bifidobacterium. However, due to the variation in test results, the method notes that it “should be used with caution when testing bifidobacteria” (43). The overall tetracycline limit of 8 μg/ml for bifidobacteria was derived from several studies that examined a variety of bifidobacterial species, resulting in a wide range of tetracycline resistances (30). However, Gueimonde et al. found unimodal resistance across 26 B. animalis subsp. lactis strains, with an average MIC of 16.9 μg/ml (18). Commercial B. animalis subsp. lactis strains Bl-04 and HN019 encompass the low level of genetic diversity within the subspecies (14–17), despite having distinct tetracycline resistances, which made them ideal subjects to locate key resistance elements. In this study, we used novel molecular strategies to confirm and further analyze the antibiotic resistance of the two strains.

The transcriptional response during subinhibitory tetracycline exposure revealed few significant changes in either strain, showing that overall functionality does not deviate from the normal metabolic processes until highly stressed. One interesting difference during the low-level exposure was the reduction of CRISPR-Cas-associated gene expression. CRISPR systems provide resistance against invasive genetic elements in bacteria (44) and have been harnessed to provide broad programmable genome editing capabilities (45). The downregulation of *cas* genes highlights the focus of the bacteria on hibernation, reducing the energy expended in nonessential pathways to survive like protection against phage predation. This supports recent findings that resistance to phage predation and antibiotics are inversely related (46). Recent efforts use endogenous CRISPR-Cas systems to modify a bacterium's own genome (47, 48); however, specificity is paramount, as self-targeting spacers can be fatal to a cell (49). With the variety of CRISPR-Cas systems in bifidobacteria and other organisms (50–52), these results suggest that concentrations of tetracycline or other antibiotics may be optimized to control nonspecific targeting and increase genome editing specificity using endogenous CRISPR in bacteria.

Acute exposure at higher concentrations of tetracycline caused substantial differences in gene expression. Protein, complex carbohydrate, and cell wall metabolisms were downregulated, notably in dTDP-rhamnose synthesis and rhamnose-containing glycans. Rhamnose polysaccharides have been previously described in B. animalis subsp. *lactis* (53) and are involved in cross-talk interaction with the host immune system (54). Moreover, they can be associated with human cell adherence and bacteriophage receptors (55). Alternatively, there was upregulation of transporter and amino acid metabolism, specifically that of methionine. This may be due to tetracycline inhibiting methionine, the typical starting amino acid in proteins, from incorporating with the ribosome. These findings are similar to other transcriptome and proteome experiments with tetracyclines (56, 57), even within a eukaryotic host (58). This is likely due to the cells entering the persistence stage, which is when cells become nondividing and dormant due to antibiotic exposure (59). Under these conditions, bacteria only perform essential functions, while simultaneously absorbing maximum amounts of environmental nutrients and exporting tetracycline. Persistence responses and thresholds are not well-studied in the dietary supplement industry, yet are important, since cells exposed to tetracycline levels above the MIC are still metabolically active.

A key metabolic pathway with consistent genetic upregulation was the pantothenate shunt, which synthesizes coenzyme A from vitamin B_5_ and is involved in metabolic reactions like lipid metabolism and respiration (60). Microbial cells uptake pantothenate, an ingredient in the ISO-Sensitest media, typically through the *panT* or *panF* transport genes (61). Although neither gene was located, pantothenate transporters are often colocalized with other pantothenate metabolic genes (62), and indeed, the *ilvC*, *panE*, and *panK* genes all were proximal to transporter genes upregulated in the LOG experiments. This indicates that environmental pantothenate and its derivative CoA are important for tetracycline tolerance. Along with being a key cofactor for many cellular processes, CoA is required to make nonribosomal peptides functional (63). Nonribosomal peptide synthesis is widespread in bacteria (64), and it could circumvent ribosome use to maintain key cellular functions during persistence. One of the genes in the pantothenate shunt was found in a cluster of upregulated genes adjacent to an rRNA operon and the putative *tet*(P) gene. This suggests that the rRNA may be directly protected due to the proximity of the *tet*(P) gene, which could then translate the adjacent CoA and transporter genes despite exposure to tetracycline.

Although the Bl-04 and HN019 genomes were considerably similar, there were several polymorphic regions, specifically indels 8 and 21, with expression differences that likely altered their phenotypes in each experiment. Small genetic differences contributing to additional tetracycline resistance are well established, as point mutations to the 16S rRNA gene can prevent tetracycline molecules from binding properly (26, 27, 65). Furthermore, an SNP in a tRNA dimethylallyltransferase (*miaA*) gene in B. animalis subsp. *lactis* was theorized to increase resistance levels (17). Indels 19 and 20, which are involved with lactose and galactose uptake, were upregulated in HN019 only during the LOG experiments. This may be beneficial, as both sugars are involved with exopolysaccharide biosynthesis and are key during stress conditions (54). Indel 15 is an SNP in the *glcU* gene that has been shown to increase glucose uptake several times over (15). If monosaccharide metabolism is preferred to disaccharide and polysaccharide metabolism during tolerance, then any increase in glucose intake would be greatly beneficial. We also noted that indel 14B altered the predicted restriction site in the gene, although the gene was not differentially expressed in our experiments. Nonetheless, epigenetic control of gene expression is common in bacteria, and epigenetic factors contributing to inherited tetracycline resistance have been demonstrated previously (66). Altogether, our results suggest that there are many genetic regions in B. animalis subsp. *lactis* besides *tet*(W) that combine to contribute to antibiotic resistance.

The *tet*(W) gene, assumed to provide resistance to tetracycline, is upregulated during the subinhibitory concentrations. Interestingly, the putative *tet*(P) in B. animalis subsp. *lactis* is a ribosomal protection GTPase similar to the *tet*(W), yet the *tet*(P) gene was upregulated during the LOG experiments, while *tet*(W) was not. The putative *tet*(36) is a tetracycline efflux pump related to genes previously reported in Bifidobacterium breve and Bifidobacterium longum (67, 68), and many other transporter genes were upregulated during tetracycline exposure. Indeed, the most apparent cause of additional resistance in HN019 is the *emrB* multidrug efflux gene, which is likely affected by indel 21 in the flanking promoter. Enzymatic protection genes, such as NADPH-requiring oxidoreductase *tet*(X), have not been reported in bifidobacteria (28). However, there were over 800 genes in the B. animalis subsp. *lactis* genomes, nearly 300 of which were upregulated in the LOG experiments, that were not assigned to a metabolic category, and novel antibiotic resistance genes in bifidobacteria are constantly being identified (69). Thus, there are many factors yet to be discovered that may also contribute broadly to antibiotic resistance in food and probiotic microorganisms.

Overall, we have shown that B. animalis subsp. *lactis* resistance to tetracycline does not just hinge on the *tet*(W) gene, but rather encompasses an orchestrated transcriptional network distributed across several genomic loci, in which even small polymorphisms may dramatically increase survival. Traditional microbiological methods have served to assess resistance in the past, but novel molecular assays and high-throughput nucleic acid sequencing provide a much greater resolution and should be used routinely to monitor and ensure safety for food and dietary supplement organisms.

## MATERIALS AND METHODS

### Bacterial cultures.

Vials of B. animalis subsp. *lactis* strain Bl-04 (ATCC SD-5219) and strain HN019 (ATCC SD-5674) culture were obtained from the DuPont global culture collection. Growth was generally obtained using De Man-Rogosa-Sharpe broth (part number [p/n] 288110; BD, Franklin Lakes, NJ) supplemented with cysteine-HCl (p/n C7880; Sigma-Aldrich, St. Louis, MO) (MRSC) at 0.05% (vol/vol) diluted in water (p/n 51200; Rockland, ME). Agar (p/n 214010; BD) was added to the MRSC broth as directed. The MIC testing also required ISO-Sensitest media (p/n CM0473; Oxoid, Basingstoke, Hampshire, UK). All samples in both media were grown at 37°C ± 1°C under anaerobic conditions using GasPacks (p/n 260001; BD).

### MIC testing.

Initial tetracycline trials followed the ISO method (43). Briefly, strains were streaked for isolation using a three-phase pattern on MRSC and agar plates and incubated 16 to 24 h as described above. A solution of sterile lactic acid bacteria susceptibility test medium (LSM) broth (90% ISO-Sensitest [IST] broth and 10% MRSC) was aliquoted into 3-ml glass tubes suitable for spectrophotometry. Colonies from the streak plates were picked with sterile loops and transferred into the broth to an optical density (OD) of 0.16 to 0.20 at 625 nm (Genesys 20; Thermo). The inoculum was diluted 1:500 in LSM media prior to inoculation into the antibiotics.

Tetracycline-HCl (p/n T4062-5G; Sigma-Aldrich) was diluted in molecular-grade water to 5,120 μg/ml, based on the concentration per mg on the label. The stock solution was diluted 1:10 (vol/vol) and the dilution scheme in the ISO method was then used to make 50% serial dilutions (512 μg/ml, 256 μg/ml, 128 μg/ml, etc.) to 1 μg/ml. The stock solution and dilutions were made fresh with each experiment. The dilutions were added to the inoculum at equal amounts, thus halving the effective dilutions to a range of 256 μg/ml to 0.5 μg/ml. The combination antibiotic and inoculum were transferred to a clear PCR plate (p/n 14230232; Thermo) and sealed (p/n AB0558; Thermo). Plates were incubated for 48 h at 37°C ± 1°C in anaerobic conditions before being inspected for cellular material under a magnifying glass.

To test acute exposure, the inoculum described above was not finally diluted 1:500 but was added directly to the tetracycline gradient and only incubated for 16 h to remain in mid-log phase.

### GlowMax Discover.

Concentrations of ATP were tested with the BacTiter-Glo microbial cell viability assay (p/n G8230; Promega, Madison, WI) on the GlowMax Discover microplate reader (p/n GM3000, Promega) following the manufacturer's instruction. Briefly, BacTiter-Glo buffer was added to the substrate and equilibrated to room temperature (22°C to 25°C) before use. The reagent was then added in equal parts to the samples in a black, opaque 96-well plate (p/n 3915; Costar, Corning, NY). The plate was placed on the GlowMax Discover, which was preprogrammed to mix with an orbital shaker, incubate at room temperature for 5 min, and then read the luminescence. Data were generated in relative luminescence units (RLU), which were not back calculated to ATP concentrations, although a standard of 10 mM rATP (p/n P113B; Promega) was added to each run to ensure run-to-run consistency. All samples were tested in duplicate, except for the retest that had six replicates per sample, and statistical comparisons were made using Prism v. 7.04 (GraphPad, La Jolla, CA).

The tetracycline standard dilutions were compared to the standards in the MaxSignal Tetracycline enzyme-limited immunosorbent assay (ELISA) kit (p/n 1016-04E; Bioo Scientific Corp., Austin, TX). The manufacturer's instructions for detection in milk/soured milk were used. The GlowMax instrument was programmed to make absorbance measurements of 450 nm (primary) and 600 nm (differential).

### Droplet digital PCR.

Bl-04 and HN019 samples were diluted 1:10 by adding 120 μl of sample to 1,080 μl of Butterfield's phosphate buffer (p/n R23701; Fisher, Hampton, NH) in clear 1.5-ml centrifuge tubes (Fisher). Samples were then treated with the viability dye Pemax (p/n 4900013150; GenIUL, Barcelona, Spain) to nullify DNA associated with dead cells. A 500 μM stock solution of Pemax was created by adding 100 μl of standard buffer (p/n: 4900018000; GenIUL) to a monodose vial of Pemax. Four μl (final conc. 1.67 μM) and 12 μl (final concentration, 5 μM) of Pemax solution were added to Bl-04 and HN019 tubes, respectively. Tubes were incubated for 30 min at 37°C, protected from light, and gently shaken to facilitate Pemax reaction. After incubation, viability dye was permanently bound and further reaction halted via UV light activation for 15 min on a PMA-Lite device (Biotium, Fremont, CA). Cells were lysed by transferring 1 ml of treated samples to prefilled 2.0-ml tubes containing Triple-Pure high-impact 0.1-mm zirconium beads (D1032-01; Benchmark Scientific, Edison, NJ). Tubes were placed into a solid aluminum microvial holder pretempered to −20°C and shaken for 15 min on a Mini-Beadbeater-96 (120 V; BioSpec Products, Bartlesville, OK).

A PCR mixture was created by combining reagents in the following concentrations and volumes to create 25 μl reaction volume per replicate: molecular biology-grade water, 0.42 μl (p/n SH30538.03; Thermo Fisher); 5 μM forward primer, 4.5 μl; 5 μM reverse primer, 4.5 μl; 3 μM probe 2.08 μl (IDT, Coralville, IA); ddPCR Supermix for probes (no dUTP), 12.5 μl (p/n 1863024; Bio-Rad, Pleasanton, CA); and 1 μl of treated sample. The Bl-04 probe utilized 6-carboxyfluorescein (FAM) dye, and the HN019 probe utilized 6-carboxy-2,4,4,5,7,7-hexachlorofluorescein (HEX) dye. All oligonucleotide sequences are listed in Table 2.

**TABLE 2:** Primers and probes used for the study

Name^*a*^	Sequence^*b*^
ddPCR	
Bl-04_F	5′-CTT CCC AGA AGG CCG GGT-3′
Bl-04_P	5′-6-FAM/CGA AGA TGA/ZEN/TGT CGG AAC ACA AAC ACC CGG/3IABkFQ-3′
Bl-04_R	5′-CGA GGC CAC GGT GCT CAT ATA GA-3′
HN019_F	5′-TTC GAT GGT TCG CAC AGT GA-3′
HN019_P	5-6-FAM-AAA CAG GTC/ZEN/AAT CAG CGG CGC AGG GAG/3IABkFQ-3′
HN019_R	5′-GGT CTG ATG CCG CCT GAA AT-3′
SNPs	
Indel_2_F	GCC GCA GAT CGA ATA CTG GG
Indel_2_R	AAG CGA CGA CCG AAT GCA AT
Indel_4_F	TCT GTT GCG GGA TGT CAT GC
Indel_4_R	GGC GAT TCA GGC GAA GTT CA
Indel_7_F	ACA GAA GTA GGC GAG GGG AC
Indel_7_R	AAC ATC ACC GCC GAT GAA CC
Indel_9_F	GGA ACT TGG CAG ACG TCT CG
Indel_9_R	CTG TTG ACT CCG GCT GCA TT
Indel_11_F	GCA GAT CGC CCC ATT GAA CA
Indel_11_R	TCG GGT CTG CTC GAC ATT CT
Indel_14A_F	CAC ACG ATC GGA ACC AGT GG
Indel_14A_R	ACG AAC GAA GTA GCC GAG GA
Indel_14B_F	CGC CGA ATC GCC ATA ATC CA
Indel_14B_R	CAG CCC GAA TCC ACT TGA CC
Indel_17_F	CTC AGT GTG CAC GCA CTC C
Indel_17_R	GAT GCA GGT TGA GCA AGG CG
Indel_19_F	CCT CGC TGT TTC GCT CTG AG
Indel_19_R	GGA AGG TGA CAT GCA GAC CG
Indel_20_F	AAC GAT CAT TTC CGC CAC CC
Indel_20_R	ATG CTG TTC GAT GCG TTG GT
Indel_22_F	CAT CCA CAG CAG CCA ACT CA
Indel_22_R	CCT GAA CCA GAT TGC CAC CG
Indel_23_F	TTC GTC ACT GGA TCG CAA GC
Indel_23_R	TCC AAC AAA CTC ACC GTG GC
Indel_27_F	GCT CTT CGT CTT CGC GGT AC
Indel_27_R	CCG ACA ATC TGC GGC AAT GA

a ddPCR, droplet digital PCR; SNP, single-nucleotide polymorphism.

b ZEN, ZEN quencher (IDT); IABkFQ, Iowa Black fluorescence quencher.

Reactions were transferred to ddPCR 96-well plates. Plates were sealed using PX1 PCR plate sealer and pierceable foil heat seals (Bio-Rad). Samples were then transferred to an automated droplet generator (Bio-Rad), and droplets were formed per the manufacturer's instruction. The plate containing newly formed droplets were transferred to C1000 Touch thermal cycler with a 96-deep well reaction module (Bio-Rad). Thermocycling was completed under the following conditions: 95°C for 10 min, 95°C for 30 s, and 60°C for 1 min repeated for a total of 40 cycles, followed by 98°C for 10 min, then held at 10°C until transfer to a QX200 droplet reader (Bio-Rad). Droplets were analyzed utilizing QuantaSoft Software v. 1.7. The detection thresholds were adjusted as needed to meet the following criteria: at least 10,000 total droplets read, sample concentration between 100 and 2,000 copies per μl, and no major defects noted in 1-dimensional (1D) or 2-dimensional (2D) amplitude.

### Genome sequencing and assembly.

HN019 was resequenced to generate a complete genome. Genomic DNA was prepared as described previously, except with MRSC (1). The shotgun libraries, sequencing, and assembly were carried out at the Roy J. Carver Biotechnology Center, University of Illinois at Urbana-Champaign (UIUC).

The shotgun genomic DNA libraries were constructed from 500 ng of DNA after sonication with an ME220 ultrasonicator (Covaris, Woburn, MA) to an average fragment size of 500 base pairs (bp) with the Hyper library preparation kit from (Kapa Biosystems, Roche, Basel, Switzerland). The individually barcoded libraries were amplified with 3 cycles of PCR and run on a fragment analyzer (AATI, Ankeny, IA) to confirm the absence of free primers and primer dimers and to confirm the presence of DNA of the expected size range. Libraries were pooled in equimolar concentration and size selected on a 2% agarose gel for fragments 500 bp to 800 bp in length. The pool was further quantitated by quantitative PCR on a CFX Connect real-time system (Bio-Rad, Hercules, CA).

The pooled shotgun libraries were sequenced on two MiSeq flow cells with paired-end reads 250 nucleotides in length using the v. 3 kits. The fastq read files were generated and demultiplexed with the bcl2fastq v. 2.17.1.14 conversion software (Illumina, San Diego, CA). For Nanopore sequencing, 1 μg of DNA was sheared in a g-Tube. Each fragment DNA sample was converted into a barcoded Nanopore library with the 1D Native barcoding genomic DNA kit (EXP-NBD103 and SQK-LSK108). Ten libraries were pooled and sequenced on a FLO-MIN106 R94.1 flow cell on a GridION X5 sequencer for 48 h. Base calling and barcode demultiplexing were performed with Albacore 2.17.

Initial Nanopore reads were base-called using Albacore v. 2.1.10 (Oxford Nanopore Technologies, Oxford, United Kingdom), retaining the original raw signal information in FAST5 format (for later assembly polishing) and producing sequence calls in FASTQ format for initial assembly steps. FASTQ data were initially assessed using FASTQC v. 0.11.5 (70). Reads were scanned and trimmed using the tools Porechop v. 0.2.3 (71) and seqtk, as described in Table S2A. Reads were then reassessed using FASTQC to ensure that trimming occurred and to establish sequence retention. These were used in downstream assembly steps.

Initial Illumina base calls were produced using Illumina's bcl2fastq, removing any residual sequence adapters. The resulting FASTQ data were initially assessed using FASTQC and then quality trimmed using Trimmomatic v. 0.36 (72), as described in Table S2B. Reads were then reassessed using FASTQC to ensure that any quality issues were addressed prior to use in downstream assembly steps.

A hybrid assembly was originally performed using the Unicycler assembler v 0.4.3 (73), as noted in Table S1C. QUAST (74) and MUMmer (75) were used to both generate some basic overall metrics of the assembly as well as to compare to the B. animalis subsp. *lactis* Bl-04 reference genome (Table S2D). Annotation was performed using Prokka (76; see also Table S2E) and rapid annotation by subsystem technology (RAST), using the default conditions (77, 78). Finally, Bandage (79) was utilized to assess completeness and potential assembly issues.

The Bl-04 and HN019 genomes were aligned and genetic differences were identified and characterized using progressiveMauve in Geneious (80). The indels were assessed by targeted PCR using primers in Table 2 designed by Primer3 in Geneious (81). Reaction mixtures were made as follows: molecular biology-grade water, 11 μl; 100 μM forward primer, 0.25 μl; 100 μM reverse primer, 0.25 μl; AmpliTaq Gold 360 (p/n N808024; ThermoFisher), 12.5 μl; and template, 1 μl. Template samples were prepared from processing 400 μl of the frozen culture vials with the Maxwell 16 cell DNA purification kit (p/n AS1020; Promega) on a Maxwell 16 (p/n AS2000, Promega), then diluted 1:10 (vol/vol) in 1× Tris-EDTA buffer at pH 8.0 (p/n BP2473-1; ThermoFisher). The thermocycler (MyCycler; Bio-Rad) was set as follows: step 1, 95°C for 10 min; step 2, 95°C for 30 s; step 3, 57°C for 30 s; step 4, 72°C for 1 min (with steps 2 to 4 repeated 34 cycles); step 5, 72°C for 5 min; and step 6, hold at 4°C. Amplicons were visualized using 2% agarose E-gels (p/n G601802; Thermo Fisher), purified using the Purelink PCR purification kit (p/n K310001; Thermo Fisher), and sequenced with Sanger technologies (Eurofins Genomics, Luxembourg). Resulting reads were aligned in Geneious.

The genomes for both strains were screened in Geneious v 11.0.4 (Biomatters, Auckland, New Zealand) for putative antibiotic resistance genes by comparing to known gene and protein functions in the Antibiotic Resistance Genes Database (82) and the Comprehensive Antibiotic Resistance Database (83), using previously described search parameters (1). Potential matches were considered relevant if the percent pairwise nucleotide match was >80%, the threshold for antibiotic resistance gene identity (24). Putative promoters were identified using the Neural Network Promoter Prediction online software (84).

### RNA sequencing.

Four separate growth experiments were made with Bl-04 and HN019, in duplicate 3-ml amounts. First, experiment ISO_0 followed the ISO method with 0 μg/ml tetracycline, grown until the culture reached an OD of 0.14 to 0.30 (early log phase). Second, experiment ISO_4 was conducted as experiment ISO_0, except with a final concentration of 4 μg/ml tetracycline. Third, experiment LOG_8 followed the ISO method, except for the 1:500 dilution prior to inoculation. After combining to make the OD 0.16 to 0.20 inoculum, the culture was instead added directly to 16 μg/ml tetracycline, making the final tetracycline concentration 8 μg/ml. The culture was then grown until reaching OD 0.14 to 0.30. Lastly, experiment LOG_32 was conducted as ISO_8, except with a final concentration of 32 μg/ml tetracycline.

After reaching early log phase, cells were centrifuged for 10 min at 4,063 × *g*. The supernatant was removed, and 1 ml of TRIzol (p/n 15596026; Thermo) was added. The pellet was resuspended by vortexing, and the tubes were immediately frozen at −80°C. The cell pellets were later thawed, transferred to a Lysing Matrix B 2-ml tube (p/n 116911050; MPBio, Santa Ana, CA), and disrupted using a Mini-Beadbeater. The lysate was subjected to a chloroform organic extraction, followed by purification using an RNeasy minikit (p/n 74104; Qiagen, Hilden, Germany). Quality checks and quantifications of the isolated RNA were carried out on the Agilent 2100 bioanalyzer (Agilent Technologies, Santa Clara, CA). rRNA was removed prior to library construction using a Ribo-Zero rRNA removal kit (Gram-positive bacteria) (p/n MRZGP126; Illumina). Stranded cDNA libraries were prepared using a TruSeq stranded mRNA kit (p/n 20020594; Illumina), quantitated by Agilent TapeStation, pooled equimolarly, and sequenced on one flow cell lane for 75 cycles using paired-end 75-base pair sequencing on an Illumina 2500 HiSeq rapid cluster kit v. 2 (p/n PE-402-4002; Illumina) and HiSeq rapid sequencing by synthesis (SBS) kit v. 2 (p/n FC-402-4021; Illumina).

Paired-end reads were imported and mapped to the B. animalis subsp. *lactis* Bl-04 genome using the Geneious for RNA Mapper with default settings in Geneious. The sequencing reads for all experiments and replicates of both strains are publicly available. Transcript levels in the resulting assemblies were calculated using the “calculate expression levels” function, and normalized for comparison of replicates and experiments using the “compare expression levels” function with the DESeq2 method and parametric fit type (85). The assemblies were exported as BAM files and imported into ArrayStar v. 1.2 (DNAStar, Madison, WI), processed using QSeq, and normalized by reads per kilobase of transcript per million mapped reads (RPKM). Regression analyses of the RNA data were made in ArrayStar software using the Student *t* test with false discovery rate (FDR) correction. Statistical analyses between sets of samples were analyzed using the DESeq2 method, as above. Differences in expression were considered significant if the absolute confidence (−log_10_ adjusted *P* value) was ± 1.00, and the log_2_ ratio was at ±1.00, representing a *P* value of ≤0.05 and a ≥2× fold change, respectively, after normalization. Nucleotide sequences of all significantly expressed genes were concatenated and uploaded in RAST to assign metabolic categories. Expression levels were visualized using Prism, and the volcano plots were generated using Excel 2016 (Microsoft, Redmond, WA).

### Accession number(s).

The complete genome sequence for Bl-04 is publicly available (NCBI GenBank accession number CP001515). A draft genome sequence for HN019, consisting of 28 contigs, was published previously (NCBI accession number ABOT00000000.1). The new HN019 genome sequence is available in the NCBI databaseunder accession number CP031154. The paired-end reads of the RNA sequencing experiments are also available in the NCBI database under the Gene Expression Omnibus accession number GSE117878.

## Supplementary Material

Supplemental file 1
