# Supplementary material for: Transcriptional and Functional Analysis of Bifidobacterium animalis subsp. lactis Exposure to Tetracycline
Source: Appl Environ Microbiol. 2018 Nov 15;84(23):e01999-18. doi: 10.1128/AEM.01999-18 (PMC6238047; doi:10.1128/AEM.01999-18)
Supplement: Supplemental file 1 [file zam023188878s1.pdf]

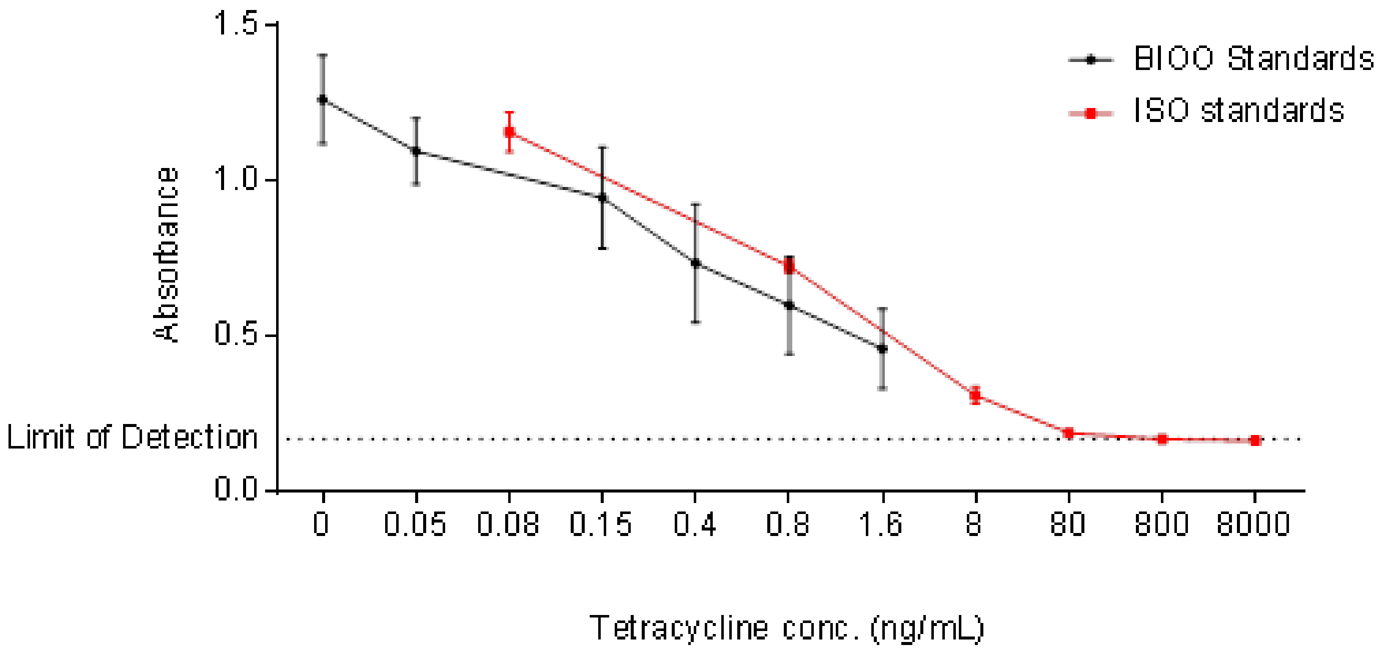

Figure S1. Validation of tetracycline standards using the BIOO MaxSignal® Tetracycline (TET) ELISA Test Kit.

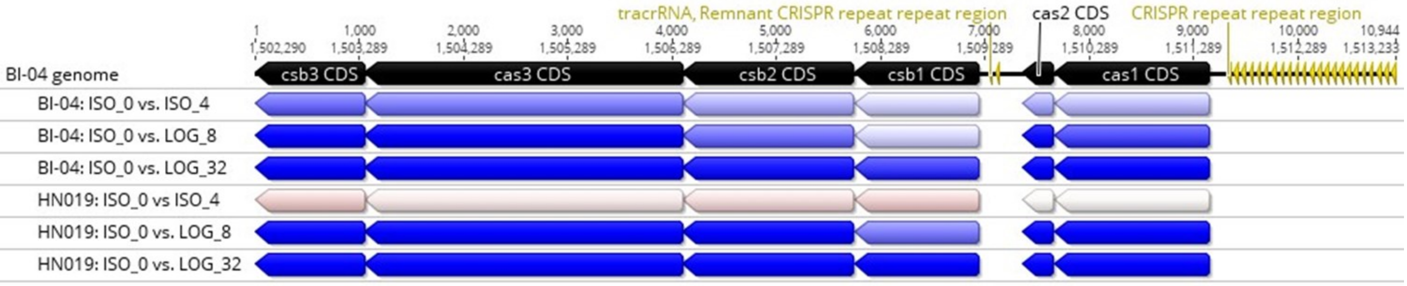

Figure S2. Transcriptional response of type I-U CRISPR system to tetracycline. The genetic region as annotated in the Bl-04 genome and colored as follows: black, coding sequences; gold, repeat regions including the tracrRNA. The arrows below each gene represent log2 ratio fold change between ISO_4, LOG_8, and LOG_32 compared to ISO_0 in descending order, with heat coloring showing downregulation and upregulation with tetracycline by blue and red, respectively.

Table S1. Polymorphisms between *B. animalis* subsp. *lactis* Bl-04 and HN019. Genes in parenthesis with indels in intergenic regions were analyzed for transcriptional differences associated with the respective indels and are visualized throughout the figures. [1]The reference number for the validation source refers to supplementary references.

Indel	Bl-04 Start Coordinate	Bl-04 ► HN019	Locus_Tag	Annotation	Impact	Validation source[1]
1	159534	(G)8 ► (G)9	BALAC_RS00705	biotin carboxylase/biotin carboxyl carrier protein	Frame Shift	[1]
2	178363	G ► T	IGR (BALAC_RS00780)	IGR (AGE family epimerase/isomerase)	-	This study
3	281373	C ► T	IGR (BALAC_RS01170)	IGR (ISL3 family transposase)	-	[1]
4	291690	(G)10 ► (G)12	IGR (BALAC_RS01215)	IGR (FAD-dependent oxidoreductase)	-	This study
5	588995	(G)4 ► (G)5	BALAC_RS02470	D-alanyl-D-alanine carboxypeptidase	Frame Shift	[1]
6	686997	A ► G	IGR (BALAC_RS02895)	IGR (S-ribosylhomocysteine lyase)	-	[1]
7	775639	G ► T	BALAC_RS03270	sensor protein	Substitution	This study
8	839425	C ► T	BALAC_RS03505	Hypothetical membrane protein	Substitution	[1]
9	871369	(G)9 ► (G)8	BALAC_RS03635	DNA repair protein *recN*	-	This study
10	903000	54 bp deletion ►	BALAC_RS03810	long-chain-fatty acid CoA ligase	Frame Shift	[1]
11	936104	C ► T	BALAC_RS03990	UTP-glucose-1-phosphate uridylyltransferase	-	This study
12	959997	A ► G	IGR (BALAC_RS04135)	IGR (PAC2 family protein)	-	[1]
13	993071	C ► T	BALAC_RS04275	hypothetical protein	Substitution	[1]
14A	1250864	C ► T	BALAC_RS05395	GTP pyrophosphokinase	Substitution	This study
14B	1252666	C ► T	BALAC_RS05395	GTP pyrophosphokinase	Substitution	This study
15	1260073	G ► C	BALAC_RS05425	glucose uptake protein *glcU*	Substitution	[1]
Mis-assembly	1304794	6138 bp deletion ►	BALAC_RS05595	Mobile element protein	-	This study
16	1456395	(G)4 ► (G)3	IGR (BALAC_RS06260)	IGR (membrane protein)	-	[1]
17	1488165	(C)10 ► (C)11	BALAC_RS08305	hypothetical protein	Frame Shift	This study
18	1512373	214 bp deletion ►	CRISPR repeat	CRISPR repeat/spacer array	-	[1]
19	1582283	C ► T	IGR (BALAC_RS06695)	IGR (galactokinase)	-	This study
20	1632627	C ► T	BALAC_RS06895	galactosyl transferase *cpsD*	Substitution	This study
21	1636887	G ► A	IGR (BALAC_RS06910)	IGR (divalent metal cation transporter MntH)	-	[1]
22	1660541	G ► T	Error in Bl-04 genome			This study
23	1677062	T ►	Error in Bl-04 genome			This study
24	1702923	► C	BALAC_RS07195	putative oxalyl-CoA decarboxylase	Frame Shift	[1]
25	1715788	54 bp deletion ►	IGR (BALAC_RS07220)	IGR (inorganic diphosphatase)	-	[1]
26	1766515	(G)8 ► (G)7	BALAC_RS07445	23S rRNA	-	N/A
27	1815298	92 bp deletion ►	Error in Bl-04 genome			This study
28A-C	1847191	8,717 bp insertion ►	BALAC_RS07800-BALAC_RS07810	NAD synthetase - hypothetical protein	-	This study
29	1881197	C ► T	BALAC_RS07840	LacI-type transcriptional regulator	-	[1]

Supplementary References

1. Briczinski EP, Loquasto JR, Barrangou R, Dudley EG, Roberts AM, Roberts RF. 2009. Strain-specific genotyping of *Bifidobacterium animalis* subsp. *lactis* by using single-nucleotide polymorphisms, insertions, and deletions. Appl Environ Microbiol 75:7501-7508.

Table S2. Scripts for assembly and annotation of *B. animalis* subsp. *lactis* HN019.

A. Quality assessment and trimming of Oxford Nanopore data

```
porechop --discard_middle \
    -i <FASTQ> --threads <CPUS> | \
    seqtk seq -L 1000 | gzip - > <SAMPLE_NAME>.qualtrim.clean.fastq.gz
```

B. Quality assessment and trimming of Illumina MiSeq data

```
java -jar trimmomatic-0.36.jar PE \
    -threads <CORES> -phred33 -trimlog <SAMPLE_NAME>.trimmomatic.log \
    <READ1> <READ2> \
    <R1>.qualtrim.paired.fastq.gz <R1>.qualtrim.unpaired.fastq.gz \
    <R2>.qualtrim.paired.fastq.gz <R2>.qualtrim.unpaired.fastq.gz \
    ILLUMINACLIP:<Tru-Seq3 ADAPTERS>:2:15:10 LEADING:28 TRAILING:28 \
    MINLEN:30
```

C. De novo Genome Assembly

```
unicycler -t <CORES> \
    -1 <TRIMMED_R1> \
    -2 <TRIMMED_R2> \
    --keep 2 \
    -l <TRIMMED_LONG_READS> -o <SAMPLE_NAME>
```

D. Quality Assessment of assemblies

```
quast.py --gene-finding \
    -o <SAMPLE_NAME> \
    -R <REFERENCE_GENOME> \
    --threads <CORES> \
    --labels <SAMPLE_NAME> \
    --scaffolds \
    -l Canu,Unicycler,SPAdes \
    <CANU ASSEMBLY> <UNICYCLER ASSEMBLY> <SPADES ASSEMBLY>
```

E. Annotation

```
prokka \
    --outdir <SAMPLE_NAME> \
    --prefix <SAMPLE_NAME> --addgenes --locustag <SAMPLE_NAME>p \
    --compliant \
    --kingdom Bacteria \
    --rfam \
    --gram ‘pos’ \
    --cpus <CORES> \
    <FINAL_ASSEMBLY>
```
